# Assuring safe artificial intelligence in critical ambulance service response: study protocol

**DOI:** 10.29045/14784726.2022.06.7.1.36

**Published:** 2022-06-01

**Authors:** Mark Sujan, Harold Thimbleby, Ibrahim Habli, Andreas Cleve, Lars Maaløe, Nigel Rees

**Affiliations:** Human Factors Everywhere Ltd. ORCID iD: https://orcid.org/0000-0001-6895-946X; Thimbleby Works; University of York; Corti ApS, Denmark; Corti ApS, Denmark; Welsh Ambulance Service NHS Trust ORCID iD: https://orcid.org/0000-0001-8799-5335

**Keywords:** artificial intelligence, emergency medical services, out-of-hospital cardiac arrest, safety

## Abstract

**Introduction:**

Early recognition of out-of-hospital cardiac arrest (OHCA) by ambulance service call centre operators is important so that cardiopulmonary resuscitation can be delivered immediately, but around 25% of OHCAs are not picked up by call centre operators. An artificial intelligence (AI) system has been developed to support call centre operators in the detection of OHCA. The study aims to (1) explore ambulance service stakeholder perceptions on the safety of OHCA AI decision support in call centres, and (2) develop a clinical safety case for the OHCA AI decision-support system.

**Methods and analysis:**

The study will be undertaken within the Welsh Ambulance Service. The study is part research and part service evaluation. The research utilises a qualitative study design based on thematic analysis of interview data. The service evaluation consists of the development of a clinical safety case based on document analysis, analysis of the AI model and its development process and informal interviews with the technology developer.

**Conclusions:**

AI presents many opportunities for ambulance services, but safety assurance requirements need to be understood. The ASSIST project will continue to explore and build the body of knowledge in this area.

## Introduction

Currently, in the United Kingdom, approximately 60,000 people sustain an out-of-hospital cardiac arrest (OHCA) annually. NHS ambulance services attempt to resuscitate around 28,000 of these people, yet survival to hospital discharge currently ranges from 2.2% to 12% (Perkins et al., 2015; Ringh et al., 2015). This represents one of the most significant challenges for reducing premature deaths. The Resuscitation Council (UK) guidelines (Perkins et al., 2015) advise that if defibrillation is delivered within 3–5 minutes of OHCA, survival rates of 50%–70% can be achieved. Improvements in survival can be made by early recognition of OHCA, quality basic life support and access to automatic external defibrillators (Ringh et al., 2015). Each minute of delay to defibrillation reduces the probability of survival by 10% (Deakin et al., 2014), hence speedy application of defibrillation and paramedic attendance on-scene are absolutely crucial.

Early recognition of OHCA by ambulance service call centre operators is important so that cardiopulmonary resuscitation can be delivered immediately (e.g. bystanders receiving telephone instructions) and delays to the arrival of ambulance crews can be minimised. However, recognition of OHCA is difficult because signs can be subtle, and the international evidence demonstrates that around 25% of OHCAs are not picked up by call centre operators (Blomberg et al., 2019).

Across health and care settings, there is an expectation that the use of artificial intelligence (AI) will transform and improve efficiency of care and patient outcomes (Topol, 2019). Examples of the use of AI include machine-learning algorithms that rely on pattern recognition, classification and prediction, such as Deep Neural Networks, which have been used in the interpretation of radiological images (Chilamkurthy et al., 2018; McKinney et al., 2020). While such image classification studies are encouraging, there is less evidence about the use of AI in pre-hospital emergency care, which has different requirements and presents its own unique set of challenges. A recent scoping review found only 16 studies looking at the use of AI in pre-hospital care settings (Kirubarajan et al., 2020), including areas such as triage of acute abdominal pain in the emergency department (Farahmand et al., 2017), early identification of patients at risk for sepsis (Giacobbe et al., 2021), risk scores in the pre-hospital setting (Spangler et al., 2019) and predicting the need for hospitalisation for paediatric asthma exacerbation (Patel et al., 2018).

However, concerns have been raised about potential negative consequences of the adoption of AI technology in healthcare (Coiera, 2018; Yu & Kohane, 2019), because the transformational reach and the effects of the widespread use of AI are hard to foresee, which can affect all aspects of healthcare including biomedical science and development (e.g. vaccines), changes to foundational business processes of how a health service is run and everyday health-related decisions people make (Coiera, 2019). Some of the challenges associated with the use of AI in healthcare are summarised in Table 1 (Challen et al., 2019; Jeter et al., 2019; Ross & Spates, 2020; Saria et al., 2018).

**Table 1.** Challenges associated with the use of AI in healthcare.

Explainability: popular AI approaches such as Deep Neural Networks produce models that are inscrutable, and hence it can be difficult to explain and justify why a particular output or decision was produced. This is also referred to as the ‘black box’ problem.
Reward hacking: AI algorithms that are trained using reinforcement learning optimise a reward function. The reward function indicates to the AI the degree of success or goodness of its decisions based on the intent specified by the designer of the AI. The AI might find ways of optimising the reward function that are unexpected and undesired – for example, an algorithm for the determination of optimal treatment strategies for septic patients might learn not to treat patients at highest risk of mortality because unsuccessful intervention attempts would not improve its reward function (Jeter et al., 2019).
Bias: AI algorithms learn from the data they are presented with, and they can learn and amplify biases in the data, such as racial bias that disadvantages specific ethnic groups.
Overfitting: AI algorithms are trained on a specific dataset, but the intention is to generalise beyond the data which the AI was trained on. However, there is a danger that the AI learns to replicate the training data exactly and fails to abstract and generalise to new data.
Overreliance: in most situations, people are still expected to provide oversight of AI decision making. However, when a system or technology performs well, people start relying on it potentially uncritically. Failures in AI decision making could go unnoticed.
Skills deterioration: people require opportunities to practise their skills. When certain tasks are automated and performed by AI, the skillsets of people are at risk of deterioration.

AI: artificial intelligence.

The focus of many studies of AI in healthcare is on the performance of the algorithms rather than on the safety and the assurance of the service within which the AI is going to be used (Sujan et al., 2019). A retrospective study from Denmark evaluated an AI system for the recognition of OHCA. The evaluation found that the AI system had significantly higher area-under-the-curve (AUC) performance and was able to recognize OHCA faster as compared to the human operators (Blomberg et al., 2019). These findings were confirmed in a subsequent Swedish study (Byrsell et al., 2021), which also looked at the use of different false positive rate thresholds. However, a recent Danish prospective study found that while the AI decision-support system was better at recognising OHCA than human operators the performance of the operator supported by the AI did not improve significantly (Blomberg et al., 2021). The study only considered outcomes (in terms of accuracy and timeliness of OHCA recognition), and it did not investigate why the joint system performance did not improve. Two reasons for this could be (1) that the algorithm’s false-positive rate threshold for this study was too high, making the operator lose confidence in the algorithm, and (2) that the recognition of this Danish EMS was struck by a ceiling effect due to their high recognition rates. There is an urgent need for further prospective studies, including small-scale evaluation studies that can build the foundation for more expensive and rigorous evaluations of AI in clinical trials (Vasey et al., 2021).

In this study, we aim to frame an AI system for the recognition of OHCA as part of the wider clinical system of the ambulance service. The aims of the study are twofold: (1) to explore ambulance service stakeholder perceptions on the safety of OHCA AI decision support in call centres, and (2) to develop a clinical safety case (Sujan et al., 2016) for an OHCA AI decision-support system. This wider clinical systems perspective of an AI decision-support system can help inform the strategy and policy for the adoption and assurance of AI technologies within ambulance services.

## Methods and analysis

The study is part research and part service evaluation. The research part utilises a qualitative study design based on thematic analysis of interview data (Braun & Clarke, 2006). The service evaluation part consists of the development of a clinical safety case based on document analysis (e.g. design and evaluation documents provided by the technology developer), analysis of the AI model (e.g. testing and inspection of the model, hazard analysis) and its development process (e.g. how training and validation data were selected, roles and qualifications of development team members), as well as informal interviews with the technology developer. The aim of a clinical safety case is to communicate in a structured way an argument, backed by appropriate evidence, as to why the system should be considered acceptably safe for use for a given task. As determining what might be regarded acceptably safe is usually a negotiation between developer and regulator, a more modest (and less contentious) aim of the clinical safety case is to make explicit the risk position and risk profile of a system (Sujan & Habli, 2021).

This study design was chosen because the qualitative research involving stakeholder interviews has the potential to provide generalisable insights into the perceptions of ambulance service staff on the safety of an AI support system, while the inclusion of the service evaluation will allow for in-depth analysis of the development process of AI technology, rather than just the evaluation of its performance. This consideration of the technology development process is an aspect that is currently missing from the existing body of literature.

### Study part A – research: stakeholder perceptions on the safety of OHCA AI decision support

#### Setting

This single-centre study will be undertaken at the Welsh Ambulance Service NHS Trust (WAST). WAST covers an area of 20,640 km, serving a population of around 3 million. Clinical contact centre staff deal with more than half a million calls every year. WAST hosts the 111 service, a 24-hour health advice and information service for the public, and the front-end call handling and clinical triage elements of the GP out-of-hours services.

#### Participants

Potential participants are WAST staff at different levels of the organisation, including call centre operators, paramedics, call centre managers, IT staff, quality improvement staff, risk managers and educators. The study will be advertised via posters (subject to COVID-19 infection prevention and control regulations) and through the Trust email. Potential participants will receive a participant information sheet to inform them about the study. We aim to undertake ca. 15–20 interviews across the participant groups. Determination of appropriate sample size in qualitative studies is a contentious issue, with many authors adopting the concept of ‘data saturation’ as stopping criterion. However, as Braun and Clarke (2021) highlight, data saturation is often poorly defined and operationalised, and might not be applicable in all applications of thematic analysis. They suggest that determination of sample size is a pragmatic choice influenced by many contextual factors. In our case, we are aiming to recruit participants across a spread of ambulance service roles while being mindful of the burden on the ambulance service regarding release of staff.

#### Data collection

We have developed a semi-structured interview template, which has been piloted with two participants. The pilot interviews will not be included in the final analysis. The topics to be covered in the interview are shown in Table 2. Due to COVID-19, we will undertake interviews over the phone. A researcher experienced in qualitative research methods will do the interviews. Participants will be asked for verbal consent to participate in the study, and we will request that they email a signed consent form. With permission, interviews will be audio recorded and transcribed verbatim. During the transcription process any identifiers will be removed. There are limitations to the extent to which anonymity can be ensured, especially in studies where the number of participants is small. Individuals with knowledge of the organisation and its staff might infer from quotations the identity of the interview participant. This risk will be communicated to participants in the information sheet. All data will be collected and stored in accordance with General Data Protection Regulations (GDPR). Recordings and transcripts will be stored on a password-protected computer, and only people involved in the project will have access to the research data. Audio recordings will be deleted once transcribed. As per the Welsh Language Act and Research Governance in Wales, all study information can be provided in Welsh, and arrangements can be made at the request of the participant to conduct the interview in Welsh. The transcript will be translated from Welsh to English for analysis.

**Table 2.** Topic guide for semi-structured interviews.

**Introduction**	Background to the study and the interview.
**Participant background**	Interviewee’s professional background and current role.
**Impact on working practices**	Interviewee’s perceptions of how working practices might be affected/changed when using AI in call centres to support recognition of OHCA.
**Clinical decision making**	Interviewee’s perceptions of how the AI should interact with people and potential levels of autonomy.
**Training**	Interviewee’s perceptions of potential training needs arising from the use of AI.
**Confidence in safety**	Interviewee’s perceptions of what would make them more or less confident about the safety of using AI.
**Incident investigation**	Interviewee’s perceptions of how incidents involving AI should be investigated.
**Organisational readiness**	Interviewee’s perceptions on the potential barriers and enablers for adopting AI in the ambulance service.

AI: artificial intelligence; OHCA: out-of-hospital cardiac arrest.

#### Data analysis

Interview transcripts will be analysed inductively and iteratively using thematic analysis (Braun & Clarke, 2006). Interviews will be read in their entirety and will then be coded using an open-coding process by a single researcher (Saldaña, 2009). During this first-cycle coding process, an analytic memo will be kept to document thoughts and ideas, and to reflect on the coding process. In meetings of the wider research team, categories will be identified through clustering of similar or related codes. Categories will be constantly compared with the data and revised until new data do not add further conceptual insights (Corbin & Strauss, 2015). Overarching themes will be identified by analysing relationships between and across categories. The analysis process will be supported by the NVivo software package for qualitative data analysis. The findings of the thematic analysis will be described at a conceptual level, supported by illustrative quotations from the transcripts.

### Study part B – service evaluation: development of a clinical safety case

#### AI technology

The system which is going to be studied has been developed by Danish technology company Corti (https://www.corti.ai/). It is an AI-powered clinical decision-support system that is able to identify important patterns in live audio. The system consists of a natural language processing (NLP) module and an OHCA recognition module. The NLP module is trained on specific languages and dialects. To date, the system has been trained on American English, Australian English, Danish and Swedish, and is in a testing phase with British English. Within the context of WAST, the system would need to be able to correctly understand Welsh as well as English. The low number of OHCA calls made in Welsh might pose problems for training the AI system, which relies on the availability of large datasets. This needs to be considered in the safety case (i.e. the technology developers need to create an argument and provide corresponding evidence that the system is able to handle such calls). The system alerts call centre operators when critical episodes are detected. The Corti system is intended to work alongside call centre operators, using machine learning to supplement their expertise with data-backed insights. The Corti system is trained on labelled past patient calls to learn salient features indicative of critical illness (supervised learning), and combines this with a database of clinical guidelines and protocols. When the call centre operator receives a call, the Corti system interface provides an interviewing platform to guide operators. The AI reacts to important cues in the conversation, and adds them in the call history together with notes from the operator. When the AI system picks up information relevant to or indicative of cardiac arrest, this is flagged up to the call centre operator, who bears responsibility for making a final decision. This represents the use scenario for this study (i.e. a person supported by the AI), but in principle other use scenarios could be investigated in the future – for example, automated decision making by the AI.

#### Clinical safety case

NHS Digital has issued two risk management standards for health information technology, which specify safety assurance requirements and practices including the development of clinical safety cases (the documents are referred to as DCB 0129 and DCB 0160, respectively). Safety cases are a common regulatory instrument, first used in the nuclear industry, and subsequently adopted across a range of safety-critical industries in the United Kingdom, such as petrochemical, military and railways (Sujan et al., 2016). In the context of health information technology, a clinical safety case forms part of a proactive safety management approach. The purpose of the clinical safety case is to communicate why a technology is deemed acceptably safe for use in a particular clinical setting. The clinical safety case contains a structured and explicit safety argument that is supported by a body of safety evidence. The argument is usually risk-based, which requires demonstration that all relevant risks have been understood and dealt with sufficiently (Habli et al., 2018). This differs from compliance-based approaches, where a technology is assumed to be safe if it can be shown that specific technical standards have been followed. The evidence can come from diverse safety management activities, such as hazard and risk analyses, design specifications, testing and empirical evaluation. As a simple analogy, the safety case can be thought of along the lines of the discussion of a research paper, which explains and critically appraises the research findings – that is, the safety case explains why the safety evidence provides sufficient confidence that a technology is acceptably safe (Sujan & Habli, 2021).

#### Development of safety assurance argument for clinical safety case

The process for developing the above safety argument and procuring the relevant safety evidence consists of three steps:

1. A hazard analysis and risk assessment will be undertaken. A high-level risk-based safety argument will be developed based on addressing the identified risks and resultant safety requirements (Habli et al., 2020).
2. The safety requirements at the clinical system level will be refined into concrete safety requirements for the AI system (i.e. the machine learning components as well as the user interface).
3. The system will be subjected to functional testing, validation and evaluation, including quantitative outcomes time-to-recognition and time-to-action, and qualitative outcomes user experience and subjective workload. These activities will be undertaken by the research team in a simulated environment – that is, the AI system will not be integrated into live ambulance service operations.

## Acknowledgements

The authors would like to thank for their support of this project: stakeholder representatives from the College of Paramedics (UK), National Ambulance Service Medical Directors, Association of Chief Ambulance Executives and National Ambulance Research Steering Group. We would also like to thank WAST for sponsoring this study and particularly, the support of the Operations and Digital directorates.

## Author contributions

MS, NR, IH and HT developed the project idea. MS and NR drafted the first version of the study protocol. All authors reviewed and critiqued the draft protocol and contributed to subsequent versions. All authors approved the final version of the study protocol. MS acts as the guarantor for this article.

## Conflict of interest

AC, LM and ZA are employees of Corti ApS and are directly involved in the development and marketing of the AI system studied.

## Ethics

The study was approved by the Health Research Authority and Health and Care Research Wales (IRAS ref. 21/HCRW/0002). The service evaluation has been approved by the Medical and Clinical Services Directorate of the Welsh Ambulance Service NHS Trust.

The findings of the research part and the service evaluation part will be of interest to the wider pre-hospital care community as they complement current studies that focus more narrowly on the evaluation of AI on specific outcomes. We intend to engage with groups such as the Association of Chief Ambulance Executives (AACE), National Ambulance Services Medical Directors (NASMED), National Ambulance Services Research Steering Group (NARSG) and College of Paramedics (UK).

## Funding

This study received funding from the Assuring Autonomy International Programme, which is a joint initiative between the University of York and Lloyd’s Register Foundation.
